# Comparison of Effectiveness of Manual Orthodontic, Powered and Sonic Toothbrushes on Oral Hygiene of Fixed Orthodontic Patients

**DOI:** 10.5005/jp-journals-10005-1310

**Published:** 2015-09-11

**Authors:** Ruchi Sharma, Mridula Trehan, Sunil Sharma, Vikas Jharwal, Nidhi Rathore

**Affiliations:** Postgraduate Student (Third Year), Department of Orthodontics and Dentofacial Orthopedics Mahatma Gandhi Dental College and Hospital, Jaipur, Rajasthan India; Professor and Head, Department of Orthodontics and Dentofacial Orthopedics Mahatma Gandhi Dental College and Hospital, Jaipur, Rajasthan India; Professor and Head, Department of Oral and Maxillofacial Surgery, Mahatma Gandhi Dental College and Hospital, Jaipur, Rajasthan, India; Senior Lecturer, Department of Orthodontics and Dentofacial Orthopedics Mahatma Gandhi Dental College and Hospital, Jaipur, Rajasthan India; Senior Lecturer, Department of Orthodontics and Dentofacial Orthopedics Mahatma Gandhi Dental College and Hospital, Jaipur, Rajasthan India

**Keywords:** Fixed orthodontic treatment, Manual orthodontic toothbrush, Oral hygiene, Powered toothbrush, Sonic toothbrush.

## Abstract

**Introduction:** Maintenance of good oral hygiene is important for patients undergoing fixed orthodontic treatment.

**Aim:** The aim of this study was to evaluate the effectiveness of a manual orthodontic toothbrush, powered toothbrush with oscillating head and sonic toothbrush in controlling plaque, gingivitis and interdental bleeding in patients undergoing fixed orthodontic treatment, and to compare their relative efficacy.

**Materials and methods:** Sixty subjects, who were to receive orthodontic treatment with both upper and lower fixed appliances, were randomly divided into three study groups, with 20 patients in each group. Groups I to III were given manual orthodontic, powered and sonic toothbrushes, respectively. Plaque index (PI), gingival index (GI) and interdental bleeding index were scored to assess the level of plaque accumulation, gingival health and interdental bleeding at baseline; 4 and 8 weeks recall visits after fixed appliance bonding. Paired t-tests and one-way analysis of variance (ANOVA) tests were used for intragroup and intergroup comparisons. The level of statistical significance was set at p < 0.05.

**Results:** This study showed that a significant reduction in all the three indices scores was found from baseline to 4 and 8 weeks in group III. On intergroup comparison, no statistically significant differences were detected between the three groups for any of the parameters assessed.

**Conclusion:** On intragroup comparison, sonic brushes performed superiorly in reducing gingivitis, plaque and interdental bleeding as compared to the manual orthodontic and powered brushes. On intergroup comparison, the relative comparative effectiveness was found to be similar for all the three brushes.

**How to cite this article:** Sharma R, Trehan M, Sharma S, Jharwal V, Rathore N. Comparison of Effectiveness of Manual Orthodontic, Powered and Sonic Toothbrushes on Oral Hygiene of Fixed Orthodontic Patients. Int J Clin Pediatr Dent 2015;8(3):181-189.

## INTRODUCTION

Maintenance of good oral hygiene is important for patients undergoing fixed orthodontic treatment. Fixed appliance components, such as bands, brackets, wires and ligatures trap food and debris which leads to plaque accumulation. This frequently aggravates gingivitis, probing pocket depth, hyperplastic tissue, decalcification, dental caries and white spot lesions on the coronal surfaces of teeth.^1-4^ Thus, it is essential to achieve adequate plaque control in such patients.

Regular tooth brushing is advised routinely to patients undergoing fixed orthodontic therapy as a means of preventing gingival and dental disease.^5^ Since, various types of toothbrushes are available in the market with attractive appeal, there is a need for sound clinical research to evaluate their effectiveness in order to guide professional recommendations for orthodontic patients.

Numerous clinical and laboratory studies have been conducted in patients receiving fixed orthodontic treatment which compared the effectiveness of different types of manual and powered toothbrushes with conventional and advanced designs. However, the results were found to be conflicting. Therefore, this study attempts to find out the relative comparative effectiveness of various types of toothbrushes, i.e. manual orthodontic toothbrush, powered toothbrush with oscillating head and sonic toothbrush. In controlling plaque, gingivitis and interdental bleeding in patients undergoing fixed orthodontic treatment; and compare their relative efficacy by evaluating the relationship between the oral hygiene status before and during treatment.

## MATERIALS AND METHODS

This study was a randomized clinical trial carried out in the department of orthodontics and dentofacial orthopedics, Mahatma Gandhi Dental College, Jaipur.

Once the approval from the local ethical committee of the institution had been obtained, 60 systematically healthy subjects were recruited for the study and divided into the following three groups (n = 20):

*Group I:* Six males, 14 females; who were given manual orthodontic brushes, the mean age was found to be 17.9 years with a range of 13 to 25 years.

*Group II:* Eleven males, 9 females; who were given powered brushes, the mean age was found to be 20.6 years with a range of 13 to 28 years.

*Group III:* Nine males, 11 females; who were given sonic powered brushes, the mean age was found to be 19.25 years with a range of 12 to 32 years.

A written informed consent was obtained from each patient for participating in the study.

Inclusion Criteria

- Patients who were to receive fixed orthodontic treatment with upper and lower preadjusted edgewise appliance therapy simultaneously
- At least 20 teeth present in the oral cavity
- Minimum 16 brackets or bands on teeth
- Brushing habit of at least once per day
- Age between 13 and 32 years
- No use of antibiotics in the past 2 months
- Absence of menstruation or pregnancy at the time of recording scores.

Exclusion Criteria

- Presence of a systemic disease
- Use of antibiotics, steroids or nonsteroidal antiinflammatory drugs (NSAIDs) therapy within past 2 months or during the study
- Fewer than five teeth per quadrant
- Immunosuppressant drugs
- Medically compromised
- Mentally handicapped subjects
- Subjects with poor manual dexterity
- Poor compliance subjects
- Subjects who received oral hygiene instructions from dental professional in past 6 months
- Presence of severe gingival inflammation
- No obvious periodontal disease (systemic or local) or attachment loss or pocketing
- Use of antibacterial mouth rinses
- Juvenile/aggressive periodontitis
- Previous or current use of powered or manual orthodontic toothbrushes
- Gross caries lesions
- Diagnosed with early onset periodontitis
- Smoking, tobacco products
- Pregnancy
- Acute illness.

**Fig. 1:**
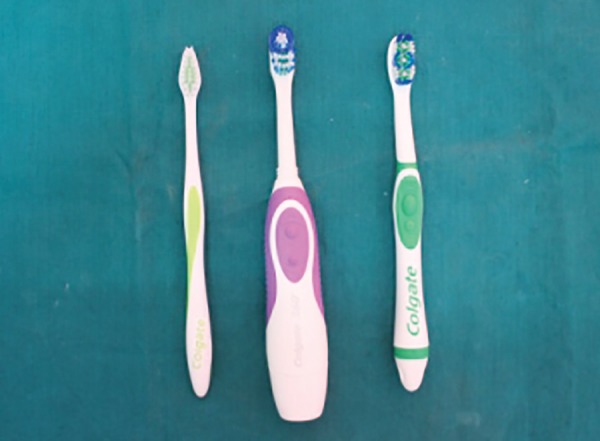
Toothbrushes used in the study: manual orthodontic, powered and sonic

The brushes evaluated in the study were (Fig. 1):

- *Manual orthodontic brush:* Colgate Ortho [Colgate Palmolive (India) ltd.]
- *Battery powered toothbrush with oscillating head:* Colgate 360° whole mouth clean [Colgate Palmolive (India) ltd.]
- *Battery powered sonic toothbrush:* Colgate 360° sonic power [Colgate Palmolive (India) ltd.]

**Fig. 2:**
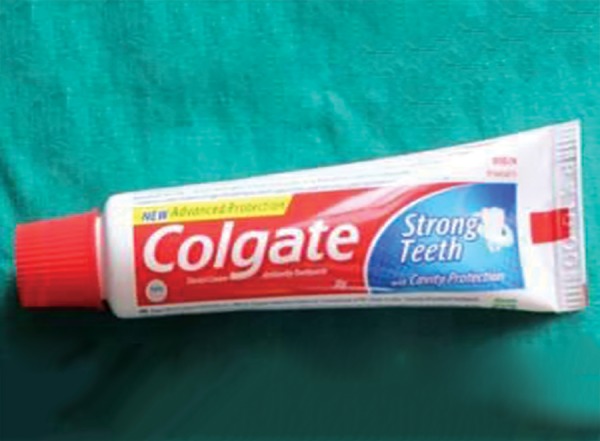
Toothpaste used in the study

**Fig. 3:**
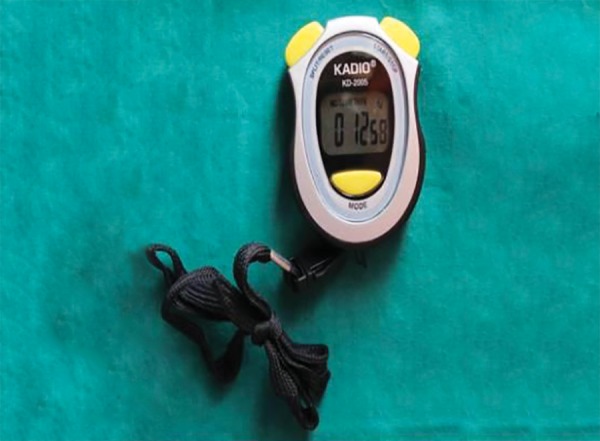
Digital timer

With these brushes, each individual was issued a fluoride-containing toothpaste (Colgate, Colgate-Palmolive (India) ltd,) free of antiplaque or anticalculus agents (Fig. 2). Oral hygiene instructions with demonstrations on a set of plastic models of the dental arches with upper and lower fixed appliances were given to all the patients. All the patients were instructed to brush twice a day for a 2-minute time period, in the morning, and before retiring at night. A digital timer (Fig. 3), a toothbrushing booklet with printed instructions and a planner were also issued to each patient. The use of interproximal brushes, dental floss, mouth rinses or any other mechanical or chemical cleaning aids was not permitted during the study.

Manual orthodontic brush with short heighted central bristles and V cut design was used by group I patients for effective cleaning of areas in and around orthodontic brackets and archwires. Patients were instructed to use their toothbrush with a combination of rolling (or sweeping) and vibration technique as advocated by Wockmock and Guay.^6^

For the powered and sonic toothbrushes, in groups II and III, the manufacturer’s recommendations were followed. The purpose and design of the toothbrush were also discussed. Patients were instructed how to place and move their powered toothbrush so as to clean one tooth at a time. Scrubbing motion of toothbrushing was used on occlusal surfaces for all the three types of toothbrushes.

After receiving oral hygiene instructions followed by professional prophylaxis (scaling and polishing), pre-adjusted edgewise fixed orthodontic labial appliances were directly bonded on all erupted teeth, except for the 1st and 2nd molars which were generally banded.

The clinical examinations were then carried out and scores for gingival, plaque and interdental bleeding indices were recorded at baseline, 4 and 8 weeks. Throughout the entire study, patients were examined by a single examiner to minimize inter-examiner bias.

Following placement of a self-retaining cheek retractor and cotton rolls, the teeth were dried with compressed air and then all the indices were recorded (Figs 4 to 11).

At baseline, plaque was assessed with the help of an explorer on the labial or buccal surfaces of the teeth, on which the fixed appliance was to be bonded, or banded using the plaque index (PI) originally described by Silness and Loe.^7^ Gingivitis was measured with a periodontal probe (Williams periodontal probe) on the labial or buccal surfaces of the teeth at baseline, 1st and 2nd visit using the gingival index (GI) described by Loe and Silness.^8^ Interdental gingival bleeding was determined by Eastman’s interdental bleeding index.^9^

**Fig. 4:**
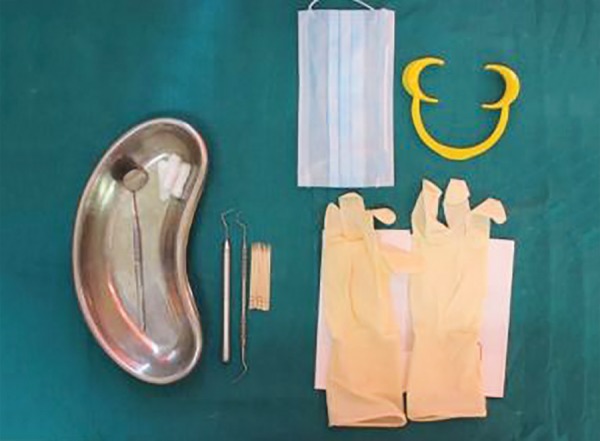
Clinical assessment tools and instruments

**Fig. 5:**
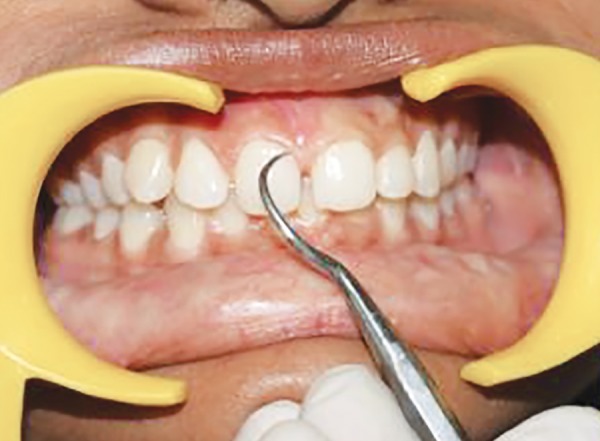
Scoring PI at mesial zone of labial tooth surface at baseline

**Fig. 6:**
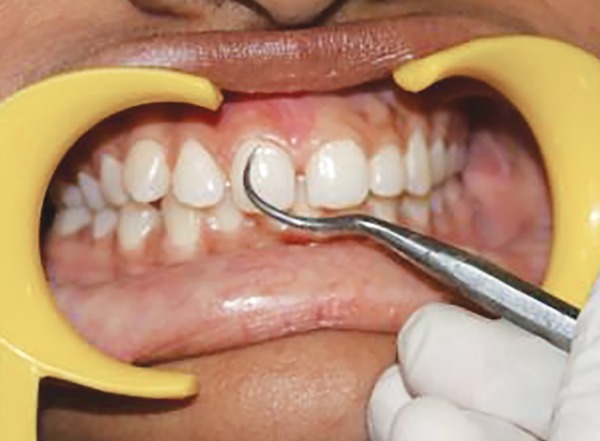
Scoring PI at middle zone of labial tooth surface at baseline

**Fig. 7:**
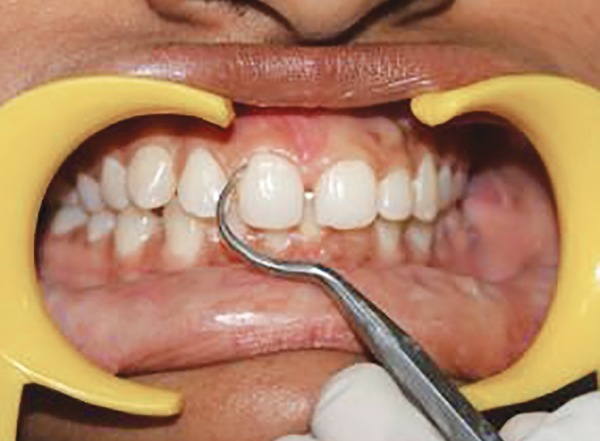
Scoring PI at distal zone of labial tooth surface at baseline

**Fig. 8:**
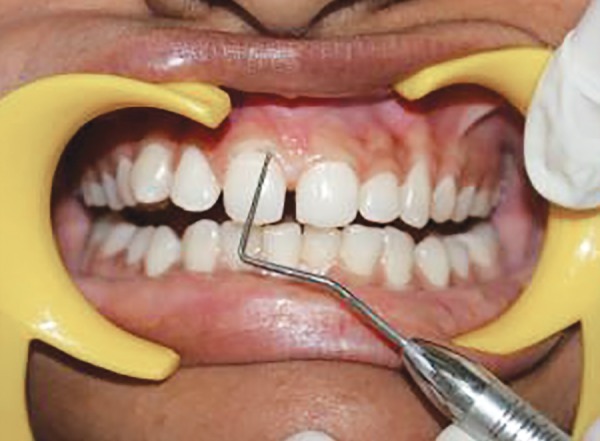
Scoring GI at mesial zone of labial tooth surface at baseline

**Fig. 9:**
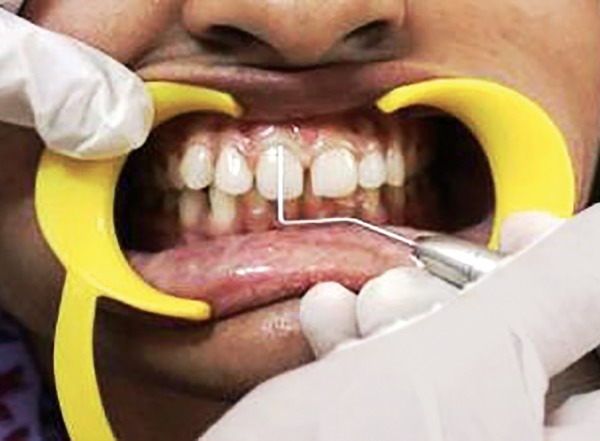
Scoring GI at middle zone of labial tooth surface at baseline

**Fig. 10:**
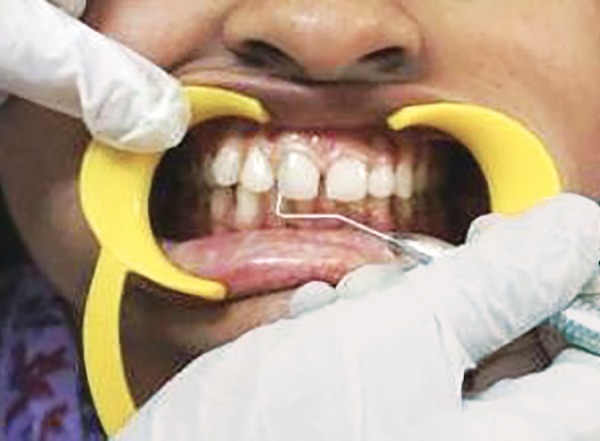
Scoring GI at distal zone of labial tooth surface at baseline

**Fig. 11:**
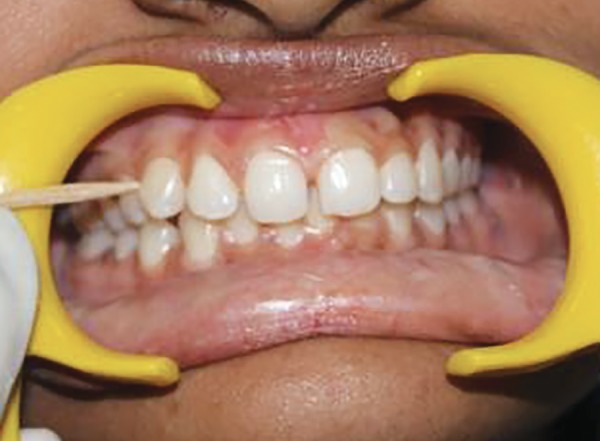
Scoring interdental bleeding index at baseline

**Fig. 12:**
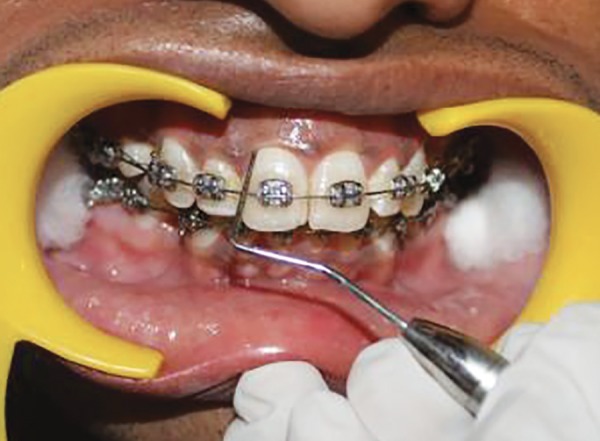
Scoring GI at distal zone of labial tooth surface at 4 and 8 weeks

**Fig. 13:**
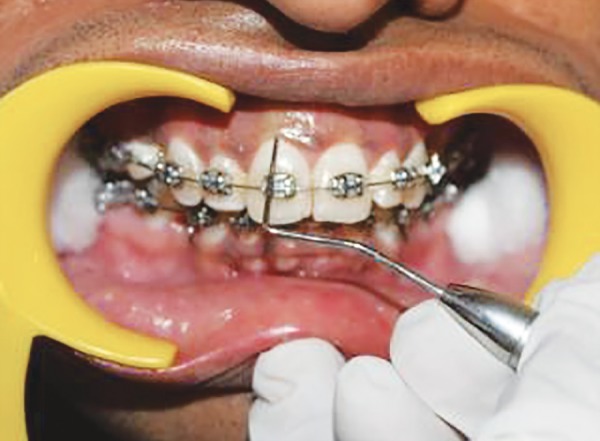
Scoring GI at middle zone of labial tooth surface at 4 and 8 weeks

**Fig. 14:**
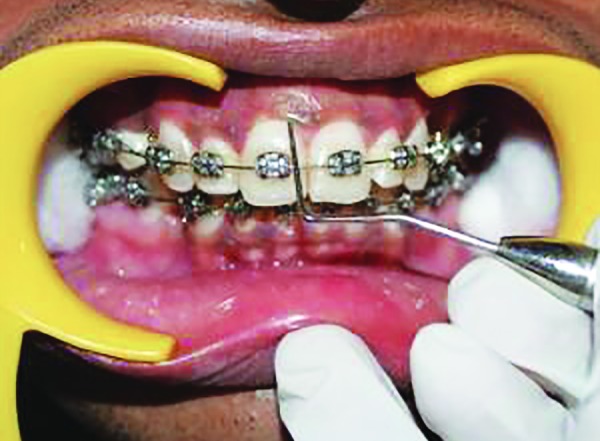
Scoring GI at mesial zone of labial tooth surface at 4 and 8 weeks

**Fig. 15:**
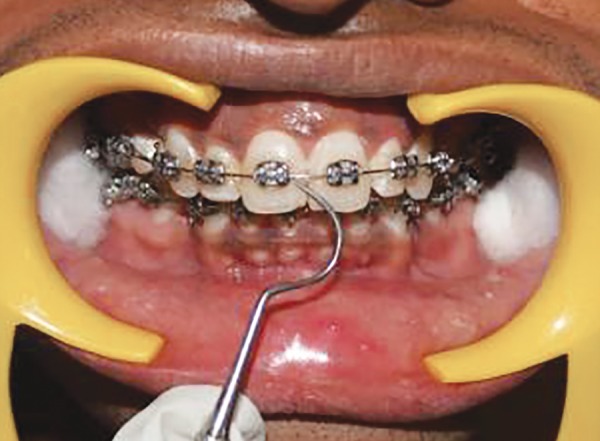
Scoring PI at zone mesial to the bracket margin at 4 and 8 weeks

**Fig. 16:**
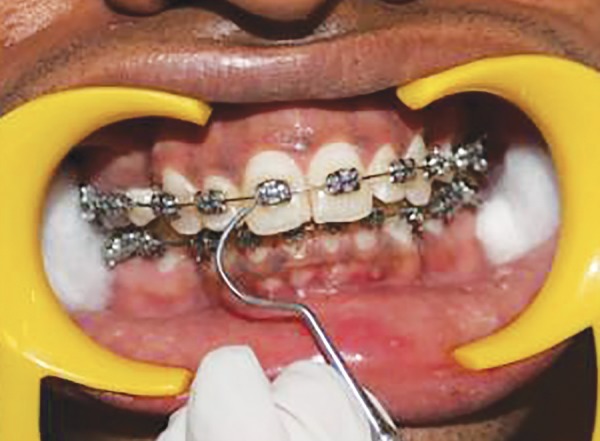
Scoring PI at zone distal to the bracket margin at 4 and 8 weeks

**Fig. 17:**
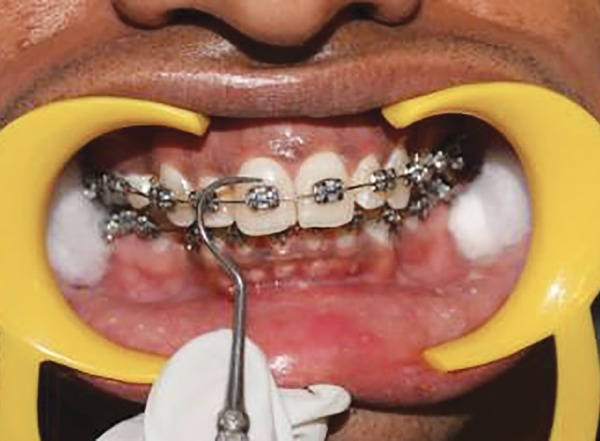
Scoring PI at zone gingival to the bracket margin at 4 and 8 weeks

**Fig. 18:**
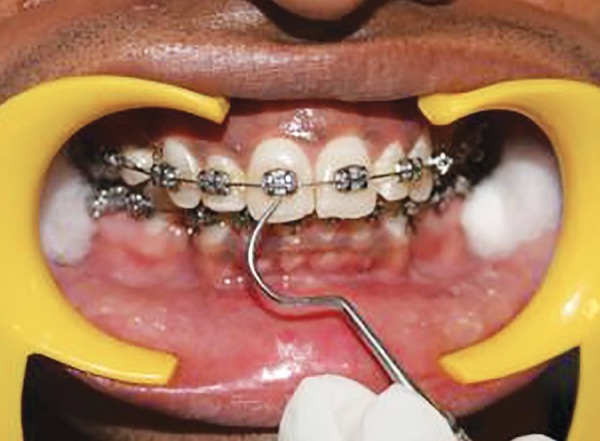
Scoring PI at zone incisal to the bracket margin at 4 and 8 weeks

**Fig. 19:**
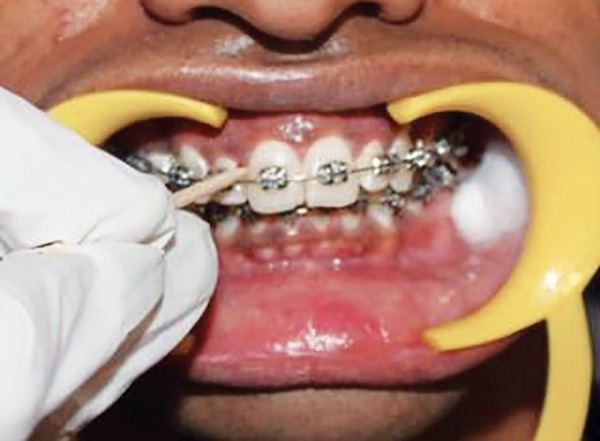
Scoring interdental bleeding index at 4 and 8 weeks

At 4 and 8 weeks recall visits after fixed orthodontic appliance bonding, patients were reassessed (Figs 12 to 19) and also questioned if they experienced any soft or hard-tissue trauma from brushing by the allocated brush. Plaque was assessed on the labial or buccal surfaces of teeth, using the orthodontic modification of the Silness and Loe PI as described by Williams et al.^10^

## STATISTICAL ANALYSIS

Statistical analysis was performed using Statistical Package for Social Sciences (SPSS) software version 17. Paired t-test was used for intragroup comparisons. One-way analysis of variance (ANOVA) was used for intergroup comparison. The level of statistical significance was set at p < 0.05.

## RESULTS

**Graph 1:**
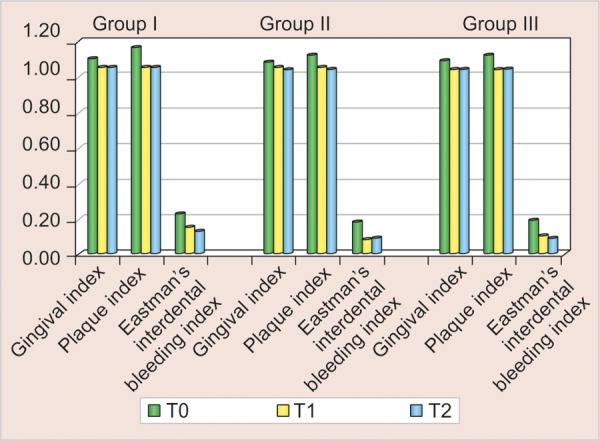
Histogram showing mean scores of all the indices from baseline to 4 and 8 weeks

The mean scores of all the three indices for all the study groups with standard deviation are shown in Table 1 and as histogram in Graph 1.

**Graph 2:**
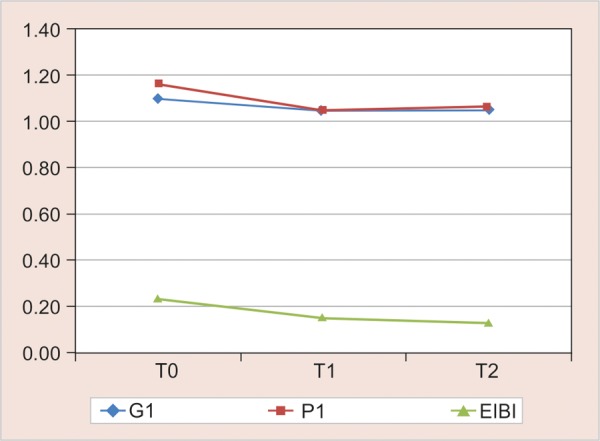
Gingival index, PI and EIBI for group I (ortho manual) from baseline to 4 and 8 weeks

**Graph 3:**
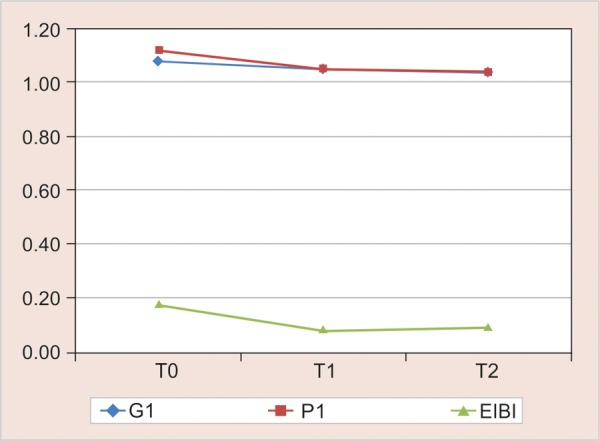
Gingival index, PI and EIBI for group II (powered) from baseline to 4 and 8 weeks

**Graph 4:**
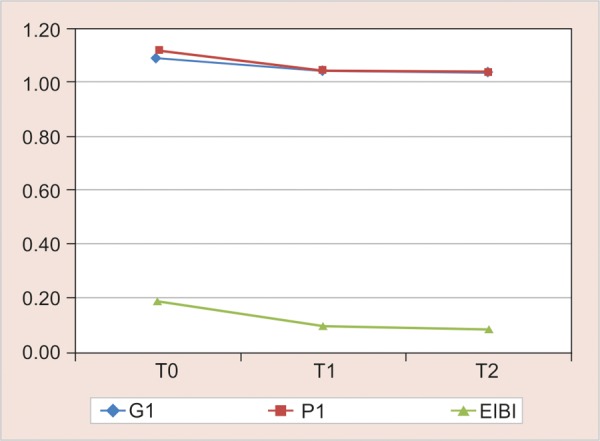
Gingival index, PI and EIBI scores for group III (sonic) from baseline to 4 and 8 weeks

The mean scores of all the three indices at baseline, 4 and 8 weeks are represented by line diagrams in Graph 2 for group I, Graph 3 for group II and Graph 4 for group III.

Intragroup comparisons of GI, PI and Eastman’s interdental bleeding index scores within each study group from baseline to 4 weeks (T0-T1), baseline to 8 weeks (T0-T2) and from 4 to 8 weeks (T1-T2) as done by paired t-test with associated p values are shown in Table 2.

Intergroups comparisons among the three study groups in mean changes from baseline to 4 weeks (T0-T1), baseline to 8 weeks (T0-T2) and from 4 to 8 weeks (T1-T2) for GI, PI and Eastman’s interdental bleeding index scores as done by one-way ANOVA with associated p-values are shown in Table 3.

**Table 1:** **Table 1:** Mean scores of all indices in each study group from baseline to 4 and 8 weeks

*Indices*		*Baseline* * (TO) mean scores* * (SD)*		*4 weeks* * (T1) mean scores* * (SD)*		*8 weeks* * (T2) mean scores* * (SD)*	
*Orthomanual*							
GI		1.10 (0.10)		1.05 (0.04)		1.05 (0.04)	
PI		1.16 (0.17)		1.05 (0.04)		1.06 (0.04)	
EIBI		0.23 (0.19)		0.15 (0.07)		0.13 (0.06)	
*Powered*							
GI		1.08 (0.16)		1.05 (0.13)		1.04 (0.13)	
PI		1.12 (0.21)		1.05 (0.13)		1.04 (0.13)	
EIBI		0.18 (0.19)		0.08 (0.07)		0.09 (0.06)	
*Sonic*							
GI		1.09 (0.19)		1.04 (0.17)		1.04 (0.17)	
PI		1.12 (0.24)		1.04 (0.17)		1.04 (0.17)	
EIBI		0.19 (0.18)		0.10 (0.07)		0.09 (0.06)	

**Table 2:** **Table 2:** Intragroup comparison for GI, PI and EIBI scores in each study group by paired t-test from baseline to 4 and 8 weeks

*Intragromp complarisons*													
*Indices*		*T0-T1*		*p-value*		*T0-T2*		*p-value*		*T1-T2*		*p-value*	
*Orthomanual*													
GI		0.05		0.02 NS		0.05		0.07 NS		0.00		0.63 NS	
PI		0.11		0.05*		0.10		0.05*		–0.01		0.78 NS	
EIBI		0.08		0.09 NS		0.10		0.03*		0.02		0.09 NS	
*Powered*													
GI		0.03		0.08 NS		0.04		0.06 NS		0.01		0.49 NS	
PI		0.07		0.01*		0.08		0.005*		0.01		0.60 NS	
EIBI		0.10		0.05*		0.09		0.15 NS		–0.01		0.47 NS	
*Sonic*													
GI		0.05		0.005*		0.05		0.02*		0.00		0.51 NS	
PI		0.08		0.04*		0.08		0.05*		0.00		0.87 NS	
EIBI		0.09		0.003*		0.10		0.002*		0.01		0.70 NS	

*p < 0.05: Statistically significant; NS: Non significant

**Table 3:** **Table 3:** Intergroup comparison for mean change in GI, PI and EIBI scores from baseline to 4 and 8 weeks among the study groups by one-way ANOVA

*Intergroup comparisons*									
*Duration*		*Orthomanual*		*Powered*		*Sonic*		*p-value*	
*Mean difference scores with SD*									
*Gingival index*									
T0-T1		0.05 (0.10)		0.03 (0.10)		0.05 (0.10)		0.77 NS	
T0-T2		0.05 (0.10)		0.04 (0.10)		0.05 (0.10)		0.94 NS	
T1-T2		0.00 (0.04)		0.01 (0.04)		0.00 (0.04)		0.66 NS	
*Plaque index*									
T0-T1		0.11 (0.24)		0.07 (0.18)		0.08 (0.18)		0.81 NS	
T0-T2		0.10 (0.23)		0.08 (0.18)		0.08 (0.18)		0.86 NS	
T1-T2		–0.01 (0.07)		0.01 (0.06)		0.00 (0.06)		0.85 NS	
*Eastman’s interdental bleeding index*									
T0-T1		0.08 (0.18)		0.10 (0.18)		0.09 (0.18)		0.94 NS	
T0-T2		0.10 (0.19)		0.09 (0.19)		0.10 (0.19)		0.98 NS	
T1-T2		0.02 (0.09)		–0.01 (0.09)		0.01 (0.09)		0.92 NS	

NS: p > 0.05, i.e. statistically not significant

## DISCUSSION

The results obtained from this study showed that in group I for whom manual orthodontic toothbrush was given, a statistically significant reduction in PI scores was found from baseline to 4 and 8 weeks (p < 0.05). No statistically significant difference was found in GI scores obtained at any point of time. This may be due to the fact that patients took time to adapt to an orthodontic toothbrush after fixed appliance bonding and the technique of placing its outer bristles at an angle of 45° to the gumline. A significant reduction in Eastmen interdental bleeding index (EIBI) was noticed at 8 weeks follow-up. This may be attributed to its longer and softer outer bristles and smaller head design than that of an ordinary or conventional medium sized manual toothbrush, which provided better interproximal cleaning.

In group II, for whom powered brushes were allocated, a statistically significant reduction in PI scores was found from baseline to 4 and 8 weeks (p < 0.05). No statistically significant reduction in GI scores was observed at any point of time. The reduction of EIBI scores from baseline to 4 weeks was found to be significant for this group which was no longer significant at 8 weeks follow-up. This may be due to the large size of the head which made it hard to maneuver into interdental areas.

In group III, for whom sonic brushes were allocated, a significant reduction in PI, GI and EIBI scores was found from baseline to 4 weeks and from baseline to 8 weeks (p < 0.05).

Grossman et al suggested that the superior performance of sonic brush may be attributed to sonic waves produced by the brush which can remove adherent bacterial plaque and disrupt bacterial growth and significantly reduce inflammation.^11^ The reason for the reductions in EIBI scores was elevated cleaning tip, extended outer bristles and tightly packed center bristles of sonic brush used in our study, which provided better interproximal cleaning with sonic vibrations.

No statistically significant differences were found in PI, GI and EIBI scores from 4 to 8 weeks for all the study groups.

On intergroup comparison, no statistically significant differences were detected among all the study groups for any of the parameters assessed like GI, PI or EIBI scores when mean differences from T0 to T1, T0 to T2 and T1 to T2 were compared. This shows that all the three toothbrushes were found to be equally effective in controling plaque, gingivitis and interdental bleeding in patients undergoing fixed orthodontic treatment.

This is in accordance with some studies where it was found that powered toothbrushes with a normal brush head or with an orthodontic brush head were as effective as manual toothbrushes in removing plaque or very few improvements with regard to plaque and in bleeding on probing were noticed with the use of powered toothbrushes as compared with manual toothbrushes.^5,12-15^ However, there are no conclusive results in the literature as per the results of reviews done by D’costa et al and Robinson et al.^16,17^

On the other hand, some studies have found powered and sonic/ultrasonic brushes to be superior over manual ones in plaque removal.^10,18-22^ Despite reduced plaque scores found in such power brush trials, improvements in the gingival health of fixed appliance patients are not very much convincing.^23^

A comparative study of use of manual and powered toothbrushes in orthodontic patients done by Borutta et al and Silvestrini et al showed a statistically significant positive variation in plaque and gingival scores with use of powered brushes.^21,24^ But, these studies were short-term trials. Sicilia et al in a review advocated that there is clear need of long-term trials in this field.^25^

In our study, no tissue trauma or any dental or gingival abrasion was noted on the gums after 8 weeks of using the powered and sonic toothbrushes. These findings confirm the safety aspects of powered toothbrushes.^26^

Another factor influencing toothbrush recommendation is cost. The price of a manual orthodontic brush is relatively less. However, powered brushes may be advantageous for certain populations that have increased dif fi-culty in maintaining oral hygiene (e.g. poor compliance patients, mentally challenged, children and younger patients, etc.).

As this study included human subjects, the influence of the ‘Hawthorne effect’ must be considered. The Hawthorne effect is expected to be greatest when the novelty device is used first. In the present study, this was not observed because scores of plaque and gingival indices at the end of 8 weeks were lower or equal than the scores at the end of 4 weeks.

## CONCLUSION

The conclusions of the present study are as follows:

- On intragroup comparison, sonic brushes performed superiorly in reducing gingivitis, plaque and interdental bleeding as compared to the manual orthodontic and powered brushes.
- On intergroup comparison, all the three toothbrushes, i.e. manual orthodontic, powered and sonic toothbrushes were found to be equally effective in controlling plaque, gingivitis and interdental bleeding in patients undergoing fixed orthodontic treatment. The relative comparative effectiveness was found to be similar for all the 3 brushes.

Thus, any of the three brushes can be recommended for orthodontic patients in order to maintain their oral hygiene during fixed orthodontic treatment. Further, a long-term follow-up throughout the orthodontic treatment can be done, to confirm the efficacy and relative effectiveness of different types of toothbrushes with various types of head designs.
